# “Many roads lead to Rome and the Artificial Intelligence only shows me one road”: an interview study on physician attitudes regarding the implementation of computerised clinical decision support systems

**DOI:** 10.1186/s12910-022-00787-8

**Published:** 2022-05-06

**Authors:** Daan Van Cauwenberge, Wim Van Biesen, Johan Decruyenaere, Tamara Leune, Sigrid Sterckx

**Affiliations:** 1 Department of Philosophy and Moral Sciences, Bioethics Institute Ghent, Ghent University, Ghent, Belgium; 2 Consortium for Justifiable Digital Healthcare, Ghent University Hospital, Ghent, Belgium; 3 Department of Nephrology, Ghent University Hospital, Ghent, Belgium; 4 Department of Intensive Care Medicine, Ghent University Hospital, Ghent, Belgium

**Keywords:** Clinical decision support systems, AI, Medicine, Automation, Division of clinical labour, Responsibility

## Abstract

Research regarding the drivers of acceptance of clinical decision support systems (CDSS) by physicians is still rather limited. The literature that does exist, however, tends to focus on problems regarding the user-friendliness of CDSS. We have performed a thematic analysis of 24 interviews with physicians concerning specific clinical case vignettes, in order to explore their underlying opinions and attitudes regarding the introduction of CDSS in clinical practice, to allow a more in-depth analysis of factors underlying (non-)acceptance of CDSS. We identified three general themes from the results. First, ‘the perceived role of the AI’, including items referring to the tasks that may properly be assigned to the CDSS according to the respondents. Second, ‘the perceived role of the physician’, referring to the aspects of clinical practice that were seen as being fundamentally ‘human’ or non-automatable. Third, ‘concerns regarding AI’, including items referring to more general issues that were raised by the respondents regarding the introduction of CDSS in general and/or in clinical medicine in particular. Apart from the overall concerns expressed by the respondents regarding user-friendliness, we will explain how our results indicate that our respondents were primarily occupied by distinguishing between parts of their job that should be automated and aspects that should be kept in human hands. We refer to this distinction as ‘the division of clinical labor.’ This division is not based on knowledge regarding AI or medicine, but rather on which parts of a physician’s job were seen by the respondents as being central to who they are as physicians and as human beings. Often the respondents’ view that certain core parts of their job ought to be shielded from automation was closely linked to claims concerning the uniqueness of medicine as a domain. Finally, although almost all respondents claimed that they highly value their final responsibility, a closer investigation of this concept suggests that their view of ‘final responsibility’ was not that demanding after all.

**Supplementary Information:**

The online version contains supplementary material available at 10.1186/s12910-022-00787-8.

## Background

Over the last decade, automated clinical decision support (CDS) using artificial intelligence (AI) has gained increasing interest. A CDS system (CDSS) is usually defined as a computer-based information system that supports decision making in patient care by integrating clinically relevant patient information and presenting it to the healthcare worker. The more widespread introduction of electronic health records (EHRs) facilitates the emergence of CDSS as it allows incorporation of clinical decision support at different levels and for different purposes. It is frequently assumed that AI-based CDSS will improve health care [1], although studies proving this hypothesis are scarce [2–4]. Moreover, the few cases where such AI-based CDSS have been incorporated in EHRs failed to improve clinically relevant outcomes [5].

More specifically, the introduction of EHR has also been associated with increased burnout in healthcare workers and decreased patient satisfaction. It has been argued that EHRs add administrative burden (the “death by a thousand clicks” [6]), and that they stand in the way of true involvement between healthcare worker and patient [7]. The implementation of CDSS into everyday care is thus considered a major step forward by some [1], and a major challenge to health care and the medical profession by others [8–10].

A lot of research has identified factors associated with acceptance of CDSS by physicians [11, 12]. Technical characteristics of the software, such as user interface and transparency, the clinical aspects of the task at hand, and the expertise of the physician with the CDS device have been reported as important factors. However, next to these engineering and technical issues, a substantial human factor remains, as the human operator’s interaction with the system is the necessary step for enacting the recommendation of the device [13]. For example, 49–96% of interruptive medication alerts are simply ignored by the operator [14]. Little evidence is available on the true underlying motivations, emotions and argumentations or their modulating factors driving the acceptance of and reaction of physicians to the incorporation of CDSS in to EHRs. Most EHRs available on the market today are designed from an administrative and informatics background perspective [7]. Consequently, they rarely consider the specific requirements of clinical tasks and the typical reasoning process of healthcare workers [15].

Therefore, we designed a mixed methods qualitative study to thematically explore the reactions and underlying reasoning of physicians when confronted with vignettes in which hypothetical CDSS incorporated in an EHR were presented. We hypothesized that many of the factors for the acceptance of CDSS by physicians reported in the literature, such as transparency, alert fatigue, and user friendliness, may have some more fundamental underlying drivers in common. Uncovering these might help to better understand, and thus potentially avoid, the sometimes ambiguous behavior of physicians when confronted with CDSS in order to enable proper development and implementation of such systems. While most researchers have focused on these more common factors, our analysis aims to uncover these more hidden underlying drivers.

## Method

### Setting and participants

This thematic analysis is part of a larger study performed in a university hospital in a transition to selecting, customizing and implementing a new electronic health record (EHR) system. While the existing EHR was considered to be “fit for the task”, it does not have a clinical decision support system (CDSS). A decision to upgrade to a more modern EHR incorporating such a CDSS was approved. All participants were thus familiar with the use of an EHR, however, their exposure to CDSS actually incorporated in an EHR was low.

Following recommendations on Q-sort methodology, we intended to interview 30 physicians, with purposive sampling to achieve a mix of gender and level of expertise (trainee, junior staff, senior staff) [16, 17]. Respondents were interviewed in a silent room, and all sources of distraction were avoided as much as possible.

### Methodological approach

We used a mixed methods approach with a Q-sort based classification of pre-defined reactions to clinical case vignettes (see Additional file 1: Qsort vignettes), in combination with a thinking-aloud approach in which reasoning and attitudes of the participant during the classification task were solicited. All sessions were done by the same interviewer (blinded for review) with expertise in the field of computerized CDSS. All sessions were audiotaped and typed out verbatim afterwards by another member of the research team (blinded for review). Q-sort techniques can be used to explore subjectivity and attitudes in a more systematic way, and to provide insights into potential patterns and concepts within this subjectivity [16]. The audiotaping of the thinking aloud during the actual completion of the Qsort allowed to not only gain insights in the perceptions of the participants, but also in their underlying reasoning, emotions and motivations.

Thirty statements describing potential actions of a (hypothetical) CDSS in four well defined clinical settings (for the vignettes describing the potential actions see Additional file 1: Qsort vignettes) were constructed. Statements represented variations of different factors already associated with uptake of CDSS by physicians: (1) transparency of the system; (2) the degree of certainty regarding the correctness of the advice provided by the device; (3) the interruption of workflow with or without obstructiveness^1^; and (4) the type of problem at hand. In a pilot, statements were evaluated for clarity and lack of ambiguity. In the first two vignettes, the focus was on decision support for medication orders, covering formulary alerts, drug-drug interaction alerts and drug-condition alerts [18]. In the third vignette, a diagnostic problem was raised, assessing automated CDS for order entry as well as for presenting and interpreting results of the ordered diagnostic radiology tests. In the last vignette, more advanced CDS for handling a complex clinical problem was presented.

### Thematic analysis

A thematic extraction of underlying opinions, attitudes and arguments of the participants was performed based on the audiotapes of the thinking-aloud during the interviews [19].

Themes and concepts issued by the participants were grouped and re-grouped by two members of the research team (blinded for review) until all concepts were placed under a non-overlapping header. Two different triangulation session with all team members were performed to reach a consensus on the thematic analysis. Quantitative results of the Qsort will be published separately.

## Results

Our findings are based on an analysis of the audio recordings of twenty-four interviews. The respondents all worked at Ghent University Hospital. Fifteen respondents identified as female and all were at different stages of their career.

Based on our analysis we identified three overarching themes, which can be further divided into smaller themes and subthemes: (1) Perceived role of the AI; (2) Perceived role of the physician; and (3) Concerns regarding AI.

The first two themes focus on the roles that were ascribed to either AI or the physician, respectively. Regardless of their general opinion towards the introduction of AI in the context of medicine, respondents were in favour of assigning certain tasks to the AI. When arguing in favour or against certain positions, the respondents almost never consciously or explicitly formulated ethical arguments, or referred to ethical principles such as justice, fairness, beneficence, or even patient autonomy.^2^ Of course this is not to say that their arguments did not reflect ethical beliefs, but rather that they did not explicitly formulate them as such. The respondents’ mostly used a two-step argumentation. First, it was, often implicitly, assumed that either the AI or the physician had a certain role to play in the medical practice. Second, it was argued that a given position was either good or bad in relation to this presupposed role. Some respondents limited the number of tasks the AI was allowed to perform by emphasizing the unique and indispensable role of the physician in clinical practice. Throughout this paper we will refer to the allocation of clinical tasks to certain actors as the division of clinical labour.

The third theme consists of concerns regarding AI that cannot be reduced to a discussion on the division of clinical labour, but that instead relates to other issues of concern. Importantly, in this last category respondents sometimes did use negative ethical statements such as ‘bad’ or ‘evil.’

### Overarching theme 1: perceived role of the AI

**Fig. 1:**
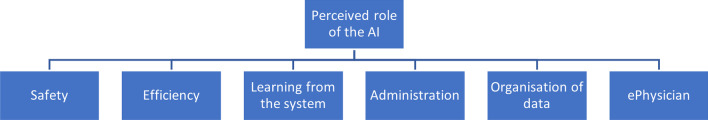
Overarching theme 1 (above), themes (below)

The first overarching theme concerns all roles that were assigned to the AI. These vary from very concrete tasks, for example all administrative tasks, to more general goals, such as increasing safety or efficiency. This first theme can be divided into six smaller themes: (1) safety; (2) efficiency; (3) learning from the system; (4) administration; (5) organisation of data; (6) and the ePhysician (Fig. 1).

### Safety

Several respondents indicated that they believed the integration of AI in clinical practice would increase safety. There were two ways in which the respondents believed AI would increase safety. First, they argued that the AI would broaden their knowledge by suggesting illnesses or therapies they would not have thought of otherwise.

AI will not replace us, but it will certainly help us. … It will increase our performance by reminding us of rare illnesses and combining all relevant data. (R9)

Second, they argued that the AI would force physicians to better reflect on their choices by implementing obstructive messages and rendering certain actions impossible. For example:

This [obstructing certain actions] could be a good thing when you are dealing with cowboys, people who think they know it best. [Obstructing them] could be a good thing to protect people against themselves. (R20)

It is dangerous when you are able to just close a pop-up without changing anything. The pop-ups should ensure that the behaviour has changed. (R4)

### Efficiency

Almost all respondents indicated they believed AI would make clinical practice more efficient. This emphasis on efficiency was especially prevalent in their judgement of the many ways in which a CDSS could be implemented. They always preferred the version that emphasized efficiency and speed in general.

I want the system to increase my efficiency and productivity. (R3)

One respondent suggested that the implementation of AI would not increase efficiency. Although the respondent initially argued that AI would make clinical practice more efficient, she later corrected her earlier statement.

I fear that our work is never done. [Even after implementing AI] I think there will be new inefficiencies. But now I am being philosophical (laughs). (R23)

### Learning from the system

Many respondents stated that they did not simply want to hand over certain tasks to the AI, but that they themselves wanted to learn from and improve their skills through the system. Most often this view was expressed by negative statements presented in the vignettes. Respondents regularly disliked certain statements, because they felt they would not ‘learn’ anything from them. For example:

I find it strange that it does not tell us why it is promoting a certain course of action. What is the origin of the decision? This way you do not learn anything [from the system]. (R9)

Generally, the respondents thought it was important that they should not just take the backseat and let the AI do the work. Some of the respondents argued that not only their job performance, but also their reasoning process should be improved by the AI.

### Administration

Many of the respondents created a dichotomy between administrative tasks on the one hand and the core medical tasks of the physician on the other. None of the respondents was very clear about the kind of tasks that belonged to these categories. Yet, almost all of them characterised the administrative tasks as being simpler and therefore easier to automate.

[Administrative tasks] are banal, they are trivial. They are very easy and should, obviously, be integrated [in the system]. If I were in charge of [ICT], this would be the first step I would take. (R6)

Indeed, most respondents had no issue with automating administrative tasks. They only expressed doubts about the automation of the tasks they perceived as being essential to their job, such as diagnosis or the prescription of therapy. Sometimes, this distinction was made very clearly:

[Unlike with medical decisions] I do trust the AI when it takes administrative decisions. Those do not look difficult to me. (R14)

### Organisation of data

Some respondents suggested that the AI would be especially useful as an organiser of data.^3^ They mostly preferred the AI to collect, organise and present data to the physician. For example, when asked which job he would most like the AI to perform, respondent 3 answered:

The best [job the AI could perform] would be the organisation of my data based on their relevance and importance. (R3)

### The ePhysician

Crucially, all the roles taken on by AI mentioned so far are merely supportive. All of the previous themes suggested that the AI and the physician should become colleagues of sorts, but they disagreed about exactly what kind of jobs the AI should perform. Moreover, some respondents indicated that they believed the AI would come to perform every task of the physician, essentially becoming an electronic physician or ePhysician:

If the system would really have all of the necessary information, I believe they would often be more reliable [than a human]. (R16)

Almost all respondents at a given point in the interview considered the prospect of their job becoming fully automated.^4^

### Overarching theme 2: perceived role of the physician

**Fig. 2:**
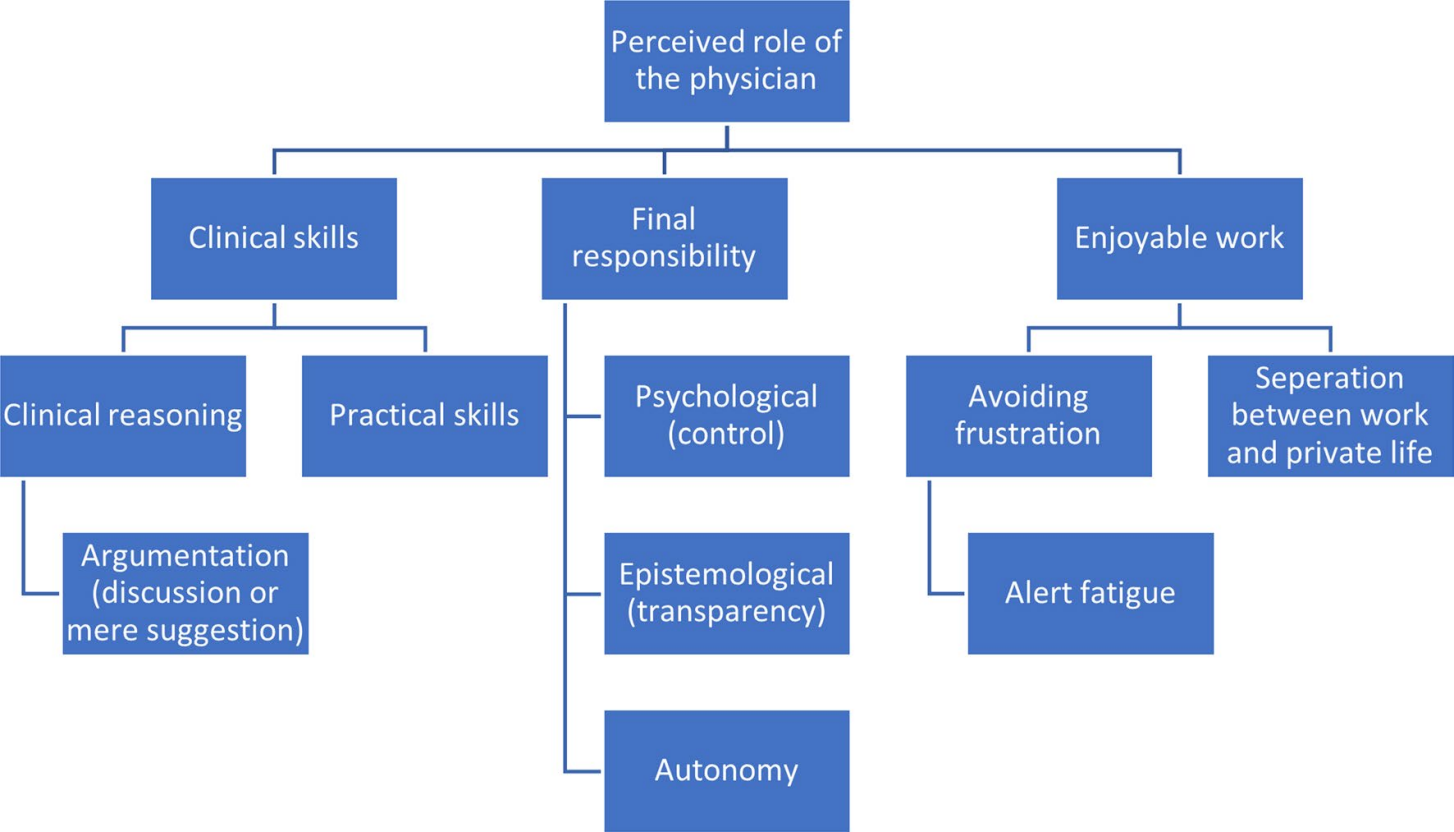
Overarching theme 2 (above), themes (middle), subthemes (below)

The second overarching theme is the counterpart of the first. It concerns all roles that are assigned to the physician. These roles are almost always framed as a reaction to the perceived encroachment of the AI. Therefore the first two themes can be seen as a confrontation of viewpoints regarding the division of clinical labour. This overarching theme can be divided into three subthemes (see Fig. 2).

### Clinical skills

Earlier we alluded to the fact that many respondents introduced a dichotomy between tasks they considered to be easier administrative tasks and those they perceived to be more difficult clinical tasks. Some respondents attempted to delineate exactly what differentiates those administrative tasks from medical ones by emphasizing certain clinical skills one needs in order to be able to perform these clinical tasks. Crucially, it was always assumed by the respondents that only humans can master these clinical skills. They identified two main clinical skills as being both crucial to the medical profession and unpossessable by the AI.

First, it was argued that it is impossible to automate clinical reasoning, but no clear reasons were provided for why this is so:

So far, I do not believe that a system exists which can fully replace the clinical reasoning process. (R16)

Some respondents, however, tried to explain which aspects of clinical reasoning process render it difficult to be automated. Those respondents expressed the view that it is difficult, if not impossible, to reduce medical reasoning to a set of rules. All of them emphasized the unique nature of every consultation. We will return to this view when discussing the third overarching theme.

Others compared the AI to one of their colleagues. Unlike the colleague, the AI is unable to explain how it came to its decision. It is at most able to give additional information, but this does not come close to a real discussion, as the respondents indicated they would have with their human colleagues. They all said that it was necessary to understand the reasoning behind a suggestion in order to accept it, but that the AI is unable to explain its position:

[With people] you are able to ask why they suggested something, they are able to give arguments for their position and I am able to react to those arguments. But here [with the AI] it ends with a suggestion. (R1)

Second, some respondents focused on certain practical skills that are essential to the medical profession. Some, for example, pointed out that interventional therapy, e.g. surgery, still has to be administered by a human physician. Of interest, they recognized that also for human physicians, differences in skills might determine which type of intervention will lead to the best result, as some physicians might have more experience with a particular technique than with others:

Many roads lead to Rome. The AI only shows me one road, but both me and my patient benefit from the road that I know best. (R20)

It was perceived by some respondents that AI does not know which therapies the physician is most familiar with. They argued the AI will sometimes suggest certain therapies, which it believes to be most efficient or well-researched, but which the physician on call does not know or is not able to administer.

### Final responsibility

Many respondents argued that the physician should have the final responsibility in clinical practice. We use the words ‘final responsibility,’ because the respondents were willing to delegate many of the physician’s tasks to the AI. Yet, the physician should always have a supervising role and, at least, every important decision should be made by him.

The reasons mentioned as to why the final responsibility should stay with the physician differed widely. Nonetheless, they can be broadly divided into three main categories. First, many respondents wanted to have final responsibility for psychological reasons. By ‘psychological’ we mean that the respondent did not mention any objective reasons, but simply wanted to stay in control:

I do not think the computer system should be allowed to block you. I have my reasons to do what I do and maybe I will think about its suggestions, but I do not want [the] IT [department] to block me at those moments. … I always want to do what I want. (R16)

Second, some respondents indicate that it is epistemologically important to stay in charge. The physician should always know what is going on. This is closely linked to the demand for more transparency because that extra information would allow the physician to stay in charge [20]:

The more information they get [from the AI], the more willing the physician will be to follow [the AI’s suggestion]. (R15)

This view differs from the previous view, in which it was stated that under no circumstance should the AI override the physician’s own opinion. If the AI is able to give adequate information on why a certain course of action has to be taken, the respondent indicates she will follow the suggestion.

Finally, some respondents emphasized that they wanted to maintain their autonomy. They wanted to be the one to take the decision, because they felt otherwise the AI would ignore their autonomy as medical professionals. Again, this is different from the two previous, because an objective reason is mentioned as to why the physician should stay in charge:

The final choice should always stay with the physician. (R11)

It is important to emphasize that these categories are not mutually exclusive. A good example of this is the following statement:

I do not want [the system] to order me around. (R1)

Respondent 1 clearly phrased his view in a way that most clearly aligns with the psychological category, but the subject matter most closely aligns with the autonomy category. This may indicate that to some respondents the lines between these different motivations are less clear.

### Enjoyable work

Finally, many respondents argued that their profession should be ‘enjoyable’. They often saw the AI as a potential negative influence on their day-to-day satisfaction at the job. Generally they all wanted to avoid unnecessary annoyance by the AI.

A recurring theme with regard to frustration was the respondents’ fear of alert fatigue. Alert fatigue refers to a situation in which people are bombarded by so many pop-ups and notifications that they become numb to their effect [9]. This results in a lot of frustration. Typical expressions of this belief are: “*Do not bother me with unimportant news*” (R5) or “*I do not want 199 notifications*” (R1).

Finally, many respondents also made clear that they worried that the introduction of AI in the workplace would further obfuscate the separation of work and private life. It was not simply that they thought the many notifications would be frustrating, but that they believed a clear separation of work and private life is a necessary precondition for an enjoyable work environment:

The border between work and private life should not become even more fluid. (R23)

### Overarching theme 3: concerns regarding AI

The first two overarching themes concerned the role of the AI and the physician in clinical practice. The third overarching theme, however, focuses on criticisms of AI that do not have anything to do with the division of clinical labour.^5^ Importantly, some of these general concerns relate to questions regarding the division of clinical labour that we identified in relation to the first and second overarching themes. However, with regard to this third main theme we will focus on the issues our respondents had with the CDSS regardless of the tasks it would be performing.

**Fig. 3:**
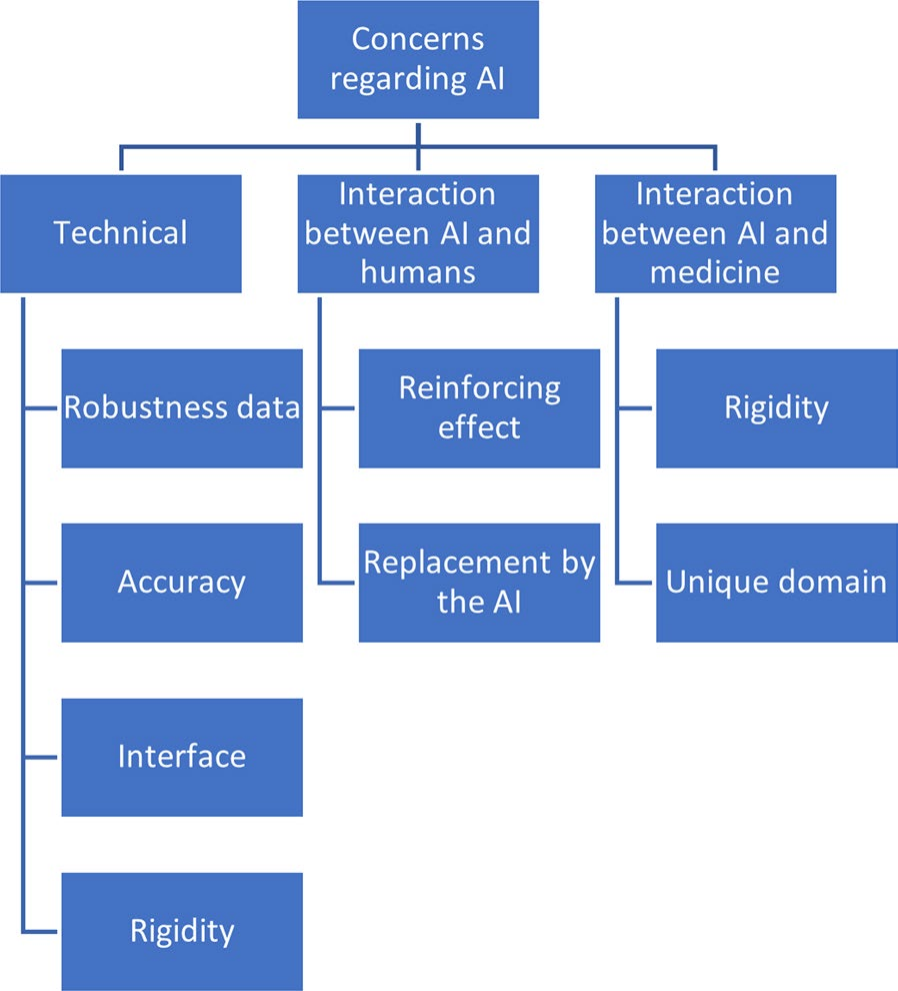
Overarching theme 3 (above), themes (middle), subthemes (below, ordered vertically)

We have subdivided these criticisms according to three perspectives. First, a purely technical perspective. Second, a perspective that focuses on the relationship between the user of the AI, in this case a physician, and the AI. Third, the perspective of the relationship between the AI and the kind of task it is being used for, in this case medical tasks. (see Fig. 3).

### Technical

Respondents were often sceptical about a variety of strictly technical aspects of the AI. We can divide these concerns into four main themes: (1) data quality; (2) accuracy of CDSS; (3) the interface of CDSS; and (4) rigidity. First, many respondents expressed doubts about the robustness of the data:

The statement [of the AI] is only as strong as the data [used by the AI] (R2)

Some respondents were more specific about why the data used by the AI could be inaccurate. It was argued that the AI would only work if it possessed all the necessary information regarding the patient, but that historically physicians have not recorded all relevant pieces of information and therefore their data would create an inaccurate picture of reality.

In order for the AI to work, you have to feed it data. But a file [on a patient made by a human physician] never contains all [of the necessary] data (R16)

A second theme concerned many respondents' worries regarding the AI’s accuracy in general. They indicated that they did not have any problem with using the AI, as long as they could be sure the AI would not make any mistakes. A more extreme version of this argument was offered by respondent 1. He argued that medicine, as a domain of research, is unable to guarantee the level of accuracy necessary for the AI to function properly.

Not only the system, but also medicine [as a field of study] has to have a certain level of accuracy [in order for these AI to function properly]. (R1)

A third issue concerned the interface of the AI and the way in which this interface could have a negative impact on clinical practice. This was one of the most common kinds of arguments given by the respondents. These concerns with the design varied widely from individual to individual. Respondent 22 said, for example, that she prefers a small amount of text, because otherwise she would not read the notification, whereas respondent 18 told us that she prefers it when the suggestions of the AI always ends with a question mark and respondent 14 told us that when she sees a button, she wants to press it. The only recurring view was that almost all respondents had a dislike for the use of red crosses to indicate that the physician had made a mistake:

The red [crosses] seem to make fun of you. I like the other [options], [because] they present you with an alternative (R5)

Finally, many respondents said they had concerns regarding the perceived ‘rigidity’ of the AI. Because this concern has to do both with the technical aspects of the AI and the way in which it relates to medicine, we will explain it below when discussing the latter.

### Interaction between AI and humans

This theme deals with concerns respondents had with the interaction between AI and human users. They broadly had two concerns: (1) reinforcement of certain attitudes; (2) habits of the human user and a fear of replacement by the AI.

First, many respondents argued that the AI could have a reinforcing effect. By reinforcement we understand a process in which pre-existing human traits are encouraged and thus made more prominent by the AI. The respondents referred to a variety of characteristics that could be encouraged by the AI. Many respondents mentioned laziness in this regard:

I mostly believe the AI will have beneficial effects, but it should not go too far. For example, alarms and help with diagnosis seem like good things, but it would be problematic if we would become too dependent on them. The danger is that we would all become very lazy and dependent. (R20)

Importantly, it is argued here that the AI would play upon pre-existing laziness. By offering quick diagnoses and alerting physicians when to act, many respondents fear that physicians would start to follow the AI unquestioningly, because that is the easy thing to do. Other respondents argued that the AI could reinforce the physician’s pre-existing intuitions or beliefs and thereby create a ‘tunnel vision’:

I find it dangerous when the system would only give you information about the illness you were already thinking about. That way the system would reinforce your first thought instead of encouraging wider reflection. … The AI must fight against tunnel vision. (R2)

We already pointed out that some respondents believed the AI would be able to completely replace the physician and that most physicians were not enthusiastic about this prospect. The second most common problem the respondents identified with AI in relation to humans was the belief that the AI would replace the human. Many respondents at some point entertained the thought of the AI, at some point, being able to completely automate the tasks of the physicians, but always immediately observed that this would be a very bad evolution.

When wondering about a future in which AI would be able to fully automate the physician’s tasks, respondent 1 said: “*In that case we [physicians] should just go home*.” When talking about a similar future, respondent 5 said: “*It is just like you [the physician] are a robot who needs to press on some buttons*.” Talking about the future of the role of AI in medicine, respondent 20 argued: “*You see that they are trying to take the decisions out of our hands*.” These reactions were often more emotional than their other responses during the interview and they often used less formal language.

Other respondents explicitly said that they do not want a future in which the AI would fully take over their job. Importantly, this refusal was not based on any criticism on performance of the AI. Rather, they argued that even if the AI was able to fully automate every aspect of the physician’s job, they still would not want it to do so:

I think it would be very dangerous when the AI would both conduct the diagnosis and prescribe the therapy all by itself. This would bypass the knowledge of the physician. We do not want the AI to do everything, otherwise the physicians could just go home. (R22)

Of note, ‘dangerous,’ at first glance seems to indicate a criticism of certain consequences of the AI, but the next sentence clarifies the respondent only criticizes the way in which the AI would replace the physician, and that it is this replacement in itself which is seen as dangerous. Most respondents experienced the automation of diagnosis and therapy as being ‘a step too far.’^6^ An exception to this rule was respondent 19, a surgeon, who believed that both diagnosis and therapy would become automated, but the automation of surgery would be “*impossible*.”

### Interaction between AI and medicine

Some people also expressed concerns regarding AI specifically because of the way it relates to the domain of medicine. This theme can be subdivided into two main concerns: (1) rigidity; (2) the idea that medicine is a ‘unique domain.’

It is believed by some that AI is too rigid, because it has to work with well-defined concepts that are measurable and mutually exclusive. This is a technical issue, because the respondents assumed this rigidity is the result of the way in which AI functions on a technical level. Yet it is also a problem in relation to clinical medicine, because the respondents believed this rigidity was a problem specifically in the context of the medical profession:

The algorithm should always leave room for doubt. In medicine it is always important to doubt. … Our domain [medicine] is very hard to automate, because it is difficult to reduce it to well-defined patterns. With us there are way too many dimensions to take into account. (R23)

This criticism closely resembles some of the views expressed by the respondents in relation to the second overarching theme, more specifically the argument that AI would not be able to automate the clinical reasoning process. These respondents seem to imply that that clinical reasoning is fundamentally non-rigid and needs to leave room for doubt.

The last issue of concern is the idea that medicine is difficult, if not impossible, to automate, because it is a ‘unique domain.’ Some respondents just seemed to take it as an assumption that medicine is fundamentally ‘different’ when it comes to automatability, but they do not substantiate this claim:

In general I am a technological optimist, but not when it comes to medicine. (R1)

The respondent does not make clear why medicine differs from other domains. It could be, because medicine is hard to reduce to a set of rules, because the data used by medical AI is rather bad or because medical AI generally makes the job of the physician less enjoyable. Yet none of these arguments are mentioned.

In general, whether the respondent saw a domain as being ‘unique’ depended on whether they were familiar with the domain. For instance, respondent 17, was more lenient towards AI that would be used to automate jobs she indicated she did not herself perform, and respondent 19, a surgeon, often emphasized the extent to which surgery is a ‘unique’ domain.

## Discussion

The results show that overall the respondents were willing to let the CDSS take over many tasks that were traditionally seen as part of the physician’s repertoire. They believed that the CDSS will make their work safer, more efficient, that they will improve their own skills by learning from the system, and that the CDSS will automate administrative and data processing tasks.

They did, however, believe that there are limits to the CDSS’ involvement in clinical practice. These limits seem to fall into three categories. First, the respondents mentioned some technical issues that need to be fixed in order for the CDSS to be ready for implementation: the data need to be robust; the system needs to be accurate; and the CDSS needs to be user-friendly. Second, they believed that some aspects of clinical practice are inherently unsusceptible to being automated. They argued that the CDSS is overly rigid or lacks certain clinical skills that are necessary in order to perform the physician’s tasks. Third, they indicated that they simply did not want specific tasks to be automated. In general, they did not give any reasons for this.

Much of the literature has focused on the technical issues with AI [20]. Based on our results we would argue, however, that these issues were not considered to be of fundamental importance by our respondents. Even if the AI would be easy to use and as accurate as possible, they indicated that they would still dislike certain parts of the physician’s job to be automated via AI. Our thematic analysis of a vignette based interview study reveals that, next to concerns that have been identified in previous research, particularly regarding user-friendliness [21, 22] and transparency [23], more in-depth psychological, epistemological and philosophical issues are at stake when physicians reflect on the introduction of CDSS. In this discussion, we would like to primarily focus on these more fundamental issues, as it is impossible to explore in-depth every theme we mentioned in the ‘Results’ section above.

### The importance of user-friendliness

Many arguments used by the respondents had something to do with the user-friendliness of the CDSS. The respondents were mostly concerned with how the system would affect their general work experience. They wanted to avoid frustration and wanted the CDSS that was best suited to complement their pre-existing work routines and habits.

Earlier research has already shown that one of the most common complaints of physicians regarding CDSS is that it is not made to suit their existing habits [24, 25]. Too often the architecture of the CDSS is such that it causes disruption of the clinical workflow, which results in it being disliked by many physicians. We believe that our respondents’ emphasis on user-friendliness should be viewed in a similar vein.

### The division of clinical labour

Respondents mostly did not use any purely ethical statements. In general, they assigned a role either to themselves or to the AI and, based on this assumption, they assessed which option best suited these preconceived roles. The respondents were not directly concerned with the ethical implications of the use itself of AI, but were mostly preoccupied with questions concerning the role of the AI in the medical profession and its relation to the physician.^7^ Central to the respondents’ argumentation was thus often a discussion on the division of medical labour.

From the results it is also apparent that most respondents adopted a dichotomy between easy, automatable ‘administrative tasks’ and difficult, non-automatable ‘clinical tasks.’ Based on our interviews it is not possible to clearly differentiate between these two categories, yet we can make some general observations about the way in which they were used.

First, the ‘medical tasks’ were implicitly assumed to be the ‘core’ of the physician’s job, while the ‘administrative tasks’ were seen as non-core tasks. Put differently, the ‘clinical tasks’ are what makes a physician a physician, according to the respondents, while the ‘administrative tasks’ were assumed to be neither necessary nor important elements of the definition of the physician’s job. Furthermore, the kinds of tasks that were assigned to each of the two categories depended on the specific job performed by the respondent (i.e. their area of medicine) and the respondent’s opinion of their job. Surgeons, for example, assigned some tasks to the non-essential category that non-surgeon respondents believed to be essential to the physician’s job. Moreover, whether or not psycho-social tasks were seen as ‘clinical’ or ‘administrative’ varied between respondents.

This dichotomy is also very important with regard to the fear of replacement expressed by most of the respondents. Generally, the respondents only felt threatened by the CDSS when it would start to be used to automate core tasks, whereas they were more positive and even enthusiastic about the automation of non-essential tasks. This supports our hypothesis that the division between clinical and administrative tasks is based mostly on personal experience of one’s job rather than on clear criteria based or their knowledge of AI. This also implies that simply taking care of some of the technical issues with the CDSS will not necessarily improve acceptance of it by physicians, because some core tasks are considered to be fundamentally off-limits when it comes to automation.

Based on this we would argue that the introduction of CDSS in the medical profession has a lot in common with the introduction of automation in other professions. Just like a factory worker has felt threatened by the existence of robots since the beginning of the twentieth century, doctors feel worried by the introduction of AI. We would like to hypothesize that the medical profession and ‘intellectual professions’ in general will experience a fear of replacement similar to the fears experienced by workers in manufacturing professions since the introduction of automation. [26] Therefore, when engaging in ethical reflection about the implementation of CDSS, we should not just consider the technical aspects of the systems in question, but also understand this as a modern labour dispute in which the physician could be seen as a threatened economic actor.

Furthermore, we would submit that automation disrupts the idea of what it means to be a physician. To many physicians medicine is not simply a job they do to earn money, but a vocation. [27] Therefore, the encroachment of AI does not just threaten the physician’s economic status, but also their self-image and the way they have chosen to spend a substantial part of their life. It is an existential issue as much as an economic issue. These existential issues should not be treated lightly as bumps on the road of progress, but should be taken seriously when contemplating whether or not to automate certain tasks.

### Claims concerning the uniqueness of medicine

Related to the previous topic is the claim that medicine is a ‘unique domain.’ For many respondents, this alleged ‘uniqueness’ of medicine was the main reason why it is impossible to completely automate the job of the physician.

As we have seen, many respondents simply stated this uniqueness as a given fact. Some respondents, however, argued that medicine is a special domain because it is fundamentally flexible, diverse or non-rigid. Put differently, they were conveying that it is not possible to reduce the practice of medicine to a certain set of ‘rules’ or to completely eradicate doubt and uncertainty. Many respondents emphasized that every case is fundamentally different and that this is crucial to understanding the complexities involved in clinical reasoning in general.

Further research should further explore and critically investigate the reasons underlying the respondents’ claims about the uniqueness of medicine [28]. For example, we believe there are certain important unique aspects of medicine that were not mentioned explicitly by the respondents and that would merit in-depth ethical, epistemological and political analysis. In their paper ‘*“Just Do Your Job”: Technology, Bureaucracy, and the Eclipse of Conscience in Contemporary Medicine*’, physicians Blythe and Curlin argue that contemporary medicine is too often understood according to a market metaphor. Hospitals are seen as businesses that provide a ‘service’ to customers. Therefore, the physicians have to become clogs in a large, anonymous, and bureaucratic machine that produces indistinguishable medical products in accordance with the will of the ‘customer.’ [27].

Building on the seminal analysis of modernity and modernisation by sociologist Max Weber in his essay ‘*Science as a Vocation*’ [29] and its interpretation by Berger, Berger and Keller [30], Blythe and Curlin consider this new understanding of medicine to be a result of the larger phenomenon of modernity spilling-over into different domains. Modernity is characterized by a ‘componential’ worldview, meaning that the world is understood as a combination of atomised components. All of these components are or should be interchangeable. In modernity people understand the world according to the principles of science and bureaucracy. Medicine too, they argue, is now often understood as a science. However, this is problematic, for:

[W]hile medicine is a practice that depends upon scientific modes of reasoning and certain features of the scientific consciousness, it is decidedly “not a science”. Rather, “it is a rational, science-using, interlevel, interpretive activity undertaken for the care of a sick person. [26]. pp. 439–440

One could argue that medicine is ‘unique’ in the sense that it is a domain that heavily depends on science and technology, but fundamentally is not a science. It is, first and foremost, a form of care. The introduction of CDSS could be seen as the next step in the ‘modernization’ of medicine and as a threat to medicine’s unique status as a heavily science-using form of ‘care’.

### The “final responsibility” of the physician

Almost all respondents argued that the final responsibility^8^ for clinical actions should stay with the (human) physician after the introduction of the CDSS in clinical practice. Earlier research has shown that this freedom or control is highly valued by users of CDSS. [24] Our analysis suggests that taking on this final responsibility is seen as one of the core roles of the human physician. This could be argued to be the main characteristic that separates a human from an electronic physician.^9^

In order to qualify as ‘having final responsibility’ the physician needs to be able to do three different things, according to our respondents. First, the physician should have the perception that (s)he is still in charge. Interestingly, the physician’s perception of control is more important than their actual control. Second, the physician should have an idea of what (s)he is doing. By this we do not mean that the physician must truly understand how the CDSS came to a recommendation, but rather that they want some general information about the CDSS’s suggestion. Third and finally, the physician wants to be able to overrule the system’s decision at all times. While the first and second criteria seem manageable to integrate into the CDSS, the third criterion is highly demanding. When implemented, the latter criterion would truly ensure that the physician would stay in charge.

However, even this last criterion should be further nuanced. Many respondents indicated that they preferred a version of the CDSS that would only let people overrule suggestions if they were able to provide a good reason for why they were doing so. A good example of this view was expressed by respondent 2:

My preferred option would be one in which the physician is able to overrule a suggestion by the AI, but only when they give the reason why they did so in order to avoid physicians making unsafe decisions.

The fact that this option was suggested by multiple respondents indicates that even this proposed ‘right to overrule’ is not as demanding as it might seem. The physician is willing to let the CDSS take over a lot of their traditional tasks. When given the chance to overrule the CDSS without any questions, they prefer the option in which the physician is obliged to substantiate a reason for wanting to overrule the CDSS. Therefore, we can conclude that the respondents actually wanted a very limited version of control to be guaranteed by the CDSS.

Furthermore, recent research has suggested that direct human supervision, as suggested by our respondents, is neither a necessary nor a sufficient criterion for being meaningfully in control. In a ground-breaking paper by Filippo Santoni de Sio and Jeroen van den Hoven, it is argued that we need to abandon the notion that humans will remain in direct control of autonomous systems in the twenty-first century. Nevertheless, they emphasise, it is important that the abandonment of direct supervision should not result in an indifference towards questions surrounding responsibility and control. Rather, they argue that we should strive towards “meaningful human control” over (semi-)autonomous AI systems, for reduced control may give rise to responsibility gaps or accountability vacuums [32]. In short, this principle of meaningful human control implies that humans, rather than computers and their algorithms, should ultimately remain in control of and thus morally responsible for relevant decisions.

They explain that the notion of meaningful human control implies that “simple human presence” or “being in the loop” is not sufficient, because “one can be present and perfectly able to influence some parts of the system by causal intervention, while (a) not being able to influence other parts of the causal chains that could come to be seen as even more relevant from a moral point of view than the parts one can in fact influence, [and] (b) not having enough information or options to influence the process”. Moreover, according to this framework of meaningful human control, “controlling in the sense of being in the position of making a substantive causal contribution to [an] activity through one’s intentional actions might not be a sufficient condition for meaningful control either, for instance, if one does not have the psychological capacity to respond appropriately under the circumstances and/or [one is] not in the position to appreciate the real capabilities of the system [one is] interacting with”. [32], p. 3.

Santoni de Sio and van den Hoven base their theory of meaningful human control on a compatibilist account of responsibility. Compatibilism refers to the philosophical belief that an actor can be in control of and responsible for an action even if she has not directly caused that action. Compatibilists believe that it is sufficient to show that the action was the result of a mental act by the actor and that the actor would have been able to act differently.

An interesting way to understand responsibility within this compatibilist framework is the concept of ‘guidance control’ proposed by John Fisher and Mark Ravizza [33]. Fisher and Ravizza claim that two conditions have to be met within a compatibilist theory in order for an actor to be morally responsible for an action. First, the decisional mechanism^10^ leading up to an action must be *responsive* to moral or factual input. It must be possible for the decision-making mechanism to *adapt* the behaviour of the actor to the relevant moral features of the situation at hand. If the actor was unable to avert the action, one cannot convincingly argue that the actor is responsible for the action in any meaningful way. Second, the actor needs to take responsibility for the decisional mechanism, meaning that the actor must be *aware* of the factual and moral *impact* of their actions.

Santoni de Sio and van den Hoven suggest that this framework is interesting to understand what would be required in order to talk of meaningful human control in the context of (semi-)autonomous systems. They identify two conditions, similar to those proposed by Fisher and Ravizza, which need to be met in order to have meaningful human control over (semi-)autonomous systems.

The first condition, *tracking*, refers to the idea that “a decision-making system should demonstrably and verifiably be responsive to the human moral reasons relevant in the circumstances… decision-making systems should track (relevant) human moral reasons.” [32], p. 7. In practice, this would mean that autonomous systems would have to be able to adjust their behaviour based on moral or factual input. Moreover, establishing *whose* moral reasons and *which* moral reasons are relevant in particular circumstances means *establishing which normative principles, norms, and values* the (semi-) autonomous system is supposed to follow or reflect.

The second condition, *tracing*, implies that “in order for a system to be under meaningful human control, its actions/states should be traceable to a proper moral understanding on the part of one or more relevant human persons who design or interact with the system.” [32], p. 9. Thus, as with the second condition of Fisher and Ravizza, it is important that *at least one person* in the design history or use context of the (semi-)autonomous system is aware of the possible impact of the system and the moral consequences of this impact. Otherwise, no one can guarantee or make sure that the system will act in accordance with moral principles.

If a (semi-)autonomous system acts in a context where both requirements are not fulfilled the system cannot be said to be under meaningful human control, according to Santoni de Sio and van den Hoven. Crucially, according to their framework, the direct supervision of a human, as proposed by our respondents, is neither a necessary nor a sufficient requirement for meaningful human control [34].

We can conclude that there is a clear divergence between this framework and the moral intuitions of our respondents regarding responsibility and control. Indeed, our respondents suggest a much less demanding view of what counts as responsibility and control. Neither the tracking nor the tracing-requirement have to be fulfilled in our respondents’ view.

First, rather than programming the system to comply with factual, ethical and legal concerns, our respondents prefer the end-user, i.e. the physician, to have the right to overrule the CDSS’ decisions. Furthermore, as we have pointed out, many respondents argued that physicians should not be allowed to overrule the AI when they have good reasons to do so and provided that they inform the AI of those reasons. Second, although our respondents indicated that they value the ability of an AI system to ‘explain’ its decisions to the physician, they did not indicate that someone in the design history of the CDSS needs to be aware of the potential factual and moral implications of the system. Rather, they emphasized that they want the end-user, i.e. the physician, to have the final responsibility for medical decisions.

## Concluding remarks

In this paper we have reported the results of a thematic analysis of twenty-four interviews with physicians. The results of this analysis were categorised into three overarching themes: the perceived role of the AI; the perceived role of the physician; and concerns regarding the AI. Each of these three main themes was divided into smaller subthemes.

Based on these themes we elaborated four important interpretations of the results. First, we argued that the respondents focussed on the way in which the AI would impact their everyday life and happiness. Second, we discussed the way in which the respondents all created a dichotomy between non-essential and core tasks of a physician. We argued that this dichotomy was linked to the likelihood of the physician in question being optimistic or not towards the automation of a given task. Third, we discussed the way in which many respondents expressed the view that medicine is a ‘unique domain’. Fourth, we explained how the desire for final responsibility was a central concern to many respondents. This demand, however, should not be understood in a strongly demanding sense.

As hypothesized, these factors were the underlying drivers of much of the discontent with the introduction of CDSS, while more common factors took a backseat or were seen as more trivial by the respondents. Although most of these common factors could be addressed by technically tweaking the CDSS, we believe that the underlying drivers that we have identified show that our respondents have fundamental issues with the automation of certain core parts of their job—regardless of how well the CDSS may perform, both from a technical and an ethical perspective. Therefore the acceptance of CDSS is not just a matter of technical improvements, but would require genuine engagement with and exploration of these underlying factors.

Based on our analysis we should like to make two recommendations for further research. First, we believe that the introduction of AI in clinical medicine should not just be studied from an ethical or a technical perspective. Indeed, our research has shown that there are important economic, social, and existential aspects to this technical transition. ‘Economic’ in the sense that a physician is an economic actor who feels threatened by the prospect of automation and whose economic interests should be taken seriously. ‘Social’ in the sense that physicians do not work in a vacuum and the social aspects of their job are important to them. ‘Existential’ in the sense that physicians are human beings who value the job they do and who want to do meaningful work.

Second, further research is needed regarding the ‘unique status’ that most respondents ascribed to the medical field. It would be interesting to explore and critically investigate the reasons underlying the respondents’ claims about the uniqueness of medicine, not only through the lens of ethics but also from an epistemological and political perspective. Such explorations could shed more light on the question as to whether or not clinical medicine really is ‘uniquely unsuited’ to being automated. We believe that both the highly variable nature of clinical problems, as emphasised by most of our respondents, and the reconceptualization of medicine as a form of ‘rational care’ in line with Blythe and Curlin [27] could be interesting perspectives to these avenues of research.

## Supplementary Information

**Additional file 1.** Qsort vignettes.

## Data Availability

All data are supplied as Additional file 1. The precise content of the vignettes is available as Additional file 1: Qsort vignettes.

## Footnotes

1 By ‘obstructiveness’ we refer to the extent to which the direct control of the operator is obstructed by the system. Obstructiveness does not necessarily mean that the operator is in no way able to override the system, but rather that their workflow is being interrupted.

2 An important exception was the use of the words ‘evil,’ ‘bad’ or ‘horrible.’ Unlike their positive counterparts, these negative statements were sometimes used by respondents. We will return to this subject when discussing the third overarching theme that emerged from our analysis.

3 The organisation of data differs from the previous subtheme. Administrative tasks are already existing tasks that the physician would like to be automated. For instance, notifying the physician when the hospital does not have a certain medicine. ‘Organising data’ on the other hand, refers to a set of tasks that did not exist before the introduction of AI and data analysis, more specifically the acquisition, organisation, and analysis of data on a scale that was not possible before the introduction of AI.

4 However, most of them reacted very negatively towards this idea. We will return to these negative reactions when reporting on the third overarching theme.

5 In the second overarching theme we already anticipated some of these criticisms, because claims that a role should be performed by a human often rested on an assumption that the AI lacked the capability to perform this task.

6 Notice how these boundaries related to the distinction made by most respondents between easy administrative and more complex clinical tasks. From this we can conclude that there is a correlation between viewing a task as being ‘automatable’ and believing a task to be fundamental to one’s job. We will return to this topic in the Discussion.

7 This may explain why the respondents were not that interested in notions that are central to ethical debates concerning AI, such as privacy.

8 We have opted to use the words ‘final responsibility’ instead of more common terms such as ‘autonomy’ or ‘control’ for multiple reasons. We believe that both ‘control’ and ‘autonomy’ indicate that one wants a great level of freedom in relation to the AI. We want to emphasise, however, that the demands of the respondents were actually rather limited. They want a level of ‘responsibility’, which we believe is not the same as control or autonomy, but only in the final stages of the decision process.

9 Although this emphasis on final responsibility may reflect views regarding legal liability [31], none of our respondents explicitly referred to legal issues and therefore we do not want to make any general claims regarding this issue. Although we consider ethical and legal questions concerning liability in contexts involving the use of AI systems to be extremely important, these questions lie beyond the scope of this paper.

10 Decisional mechanism refers to the set of causal reactions by which an action comes about. This mechanism can be mental, physical or digital. Compatibilists use this concept in order to highlight their view that free will is not a necessary precondition for holding someone responsible for an action.
