# Supplementary material for: “Many roads lead to Rome and the Artificial Intelligence only shows me one road”: an interview study on physician attitudes regarding the implementation of computerised clinical decision support systems
Source: BMC Med Ethics. 2022 May 6;23:50. doi: 10.1186/s12910-022-00787-8 (PMC9077861; doi:10.1186/s12910-022-00787-8)
Supplement: Supplementary file 1 — Additional file 1. Qsort vignettes. [file 12910_2022_787_MOESM1_ESM.pdf]

Appendix 1: Qsort vignettes

(potential statements are indicated with a number and a letter in the main text, these are here included between brackets after the statement for clarity and reference)

Vignette 1: *You want to enter in the CPOE the medication "Product E" for a new patient that has just been admitted. The CDSS produces a warning. Rank the following warnings in order of your preference and appreciation. Please speak up your thoughts while sorting the different statement.*

Qsort grid:

-2	-1	0	+1	+2
1	2	4	7	9
	3	5	8	
		6		

- Action not allowed (1A)
- Product E not available (1B)
- Product E not available; proposed alternative: Product U (1C)
- Product E not available; proposed alternative: Product U (with a link to accept this suggestion) (1D)
- Suggestion: based on anamnesis, results of imaging and lab values of this patient, Product U might be a better alternative for this patient (with a button to ignore this message) (1E)
- Suggestion: based on anamnesis, results of imaging and lab values of this patient, Product U is 95 certain to be a better alternative for this patient (this suggestion cannot be ignored) (1F)
- Suggestion: based on anamnesis, results of imaging and lab values of this patient, Product is 95% certain to be a better alternative for this patient (with a button to ignore this message and a button to select either Product E or Product U) (1G)
- Product U is a better alternative (this suggestion cannot be ignored) (1H)
- Product U is a better alternative (this suggestion can be ignored and there is a button to select either Product U or Product E) (1I)

Vignette 2: *You want to enter in the CPOE the medication "Product E" for a new patient that has just been admitted. The CDSS produces a warning. Rank the following warnings in order of your preference and appreciation. Please speak up your thoughts while sorting the different statement.*

Qsort grid:

-2	-1	+1	+2
1	2	4	6
	3	5	

- **Warning:** potential interactions with other medications this patient has been prescribed (this message cannot be ignored, no option to register the prescription of Product E) (2A)
- **Warning:** potential interactions with other medications this patient has been prescribed (this message can be ignored, if you want you can register the prescription of Product E) (2B)
- **Warning:** potential interactions with other medications this patient has been prescribed: for drug X serum levels will be increased; for drug Y there is potentialization of pro-arythmic effects (this message can be ignored, if you want you can register the prescription of Product E) (2C)
- **Warning:** potential interactions with other medications this patient has been prescribed: for drug X serum levels will be increased; for drug Y there is potentialization of pro-arythmic effects (this message cannot be ignored, you cannot register the prescription of Product E) (2D)
- **Warning:** potential interactions with other medications this patient has been prescribed: for drug X serum levels will be increased; for drug Y there is potentialization of pro-arythmic effects. Suggested better alternative: Product U (this message can be ignored, if you want you can register the prescription of Product E) (2E)
- **Warning:** potential interactions with other medications this patient has been prescribed: for drug X serum levels will be increased; for drug Y there is potentialization of pro-arythmic effects (this message can be ignored, if you want you can register the prescription of Product E, there is a button to accept Product E or Product U) (2F)

Vignette 3: A young female patients admitted to the ward for a broken leg complains about abdominal discomfort, especially in the right lower abdomen. Clinical investigation does not yield any suspicious clues. To be on the safe side, you want to order a CT-scan of the abdominal and pelvic region. *The CDSS incorporated in the Computerized Physician Ordering Entry System produces a message. Rank the following messages in order of your preference and appreciation. Please speak up your thoughts while sorting the different statement.*

Qsort grid:

-2	-1	+1	+2
1	2	4	6
	3	5	

- Request for CT scan abdominal and pelvic region in this patient denied (message on computer screen in EHR) (3A)
- Request for CT scan abdominal and pelvic region in this patient not granted as negative pregnancy test is lacking (message on computer screen in EHR) (3B)
- CT scan abdominal and pelvic region in this patient: probability of acute appendicitis less than 20% (message on computer screen in EHR) (3C)
- CT scan abdominal and pelvic region in this patient: result available, click this link (message on computer screen in EHR) (3D)
- CT scan abdominal and pelvic region in this patient: acute appendicitis (message on mobile phone) (3E)
- CT scan abdominal and pelvic region in this patient: extra-uterine pregnancy, click this link to contact obstetrician on call (message on computer screen in EHR) (3F)

Vignette 4: *During the on-call for your specialty, a patient is admitted with symptoms and complaints which you believe can be compatible with the rare syndrome XYZ. This syndrome is the domain of your colleague, who cannot be contacted, and you yourself have little experience with it. You did not manage yourself a patient with XYZ during the last 2 years.*

Classify the following statements of the CDSS integrated in your hospital EHR using Qsort

Qsirt grid

-2	-1	0	+1	+2
1	2	4	7	9
	3	5	8	
		6		

- On typing the keyword “XYZ” the box “differential diagnosis”, the CDSS produces a list of overview papers on XYZ of the last 5 years (4A)

- On typing the keyword “XYZ” the box “differential diagnosis”, the CDSS produces a link to a PDF file of the latest guideline on the management of XYZ of the European XYZ association (4B)
- Using NLP to explore your patient history and clinical examination, in combination with lab and imaging results, the CDSS produces a pop up: *“XYZ syndrome?”* (4C)
- Using NLP to explore your patient history and clinical examination, in combination with lab and imaging results, the CDSS produces a pop up: “XYZ syndrome with 70% probability” (4D)
- Using NLP to explore your patient history and clinical examination, in combination with lab and imaging results, the CDSS produces a pop up: *“XYZ syndrome?”* and produces all orders for further diagnostic work up and treatment (4E)
- Using NLP to explore your patient history and clinical examination, in combination with lab and imaging results, the CDSS produces a pop up: *“XYZ syndrome?”* with a short explanation on why this seems a plausible diagnosis. The CDSS also produces all orders for further diagnostic work up and treatment, each with a short explanation on why they might be useful. Each suggestion can be ignored or accepted. (4F)
- Using NLP to explore your patient history and clinical examination, in combination with lab and imaging results, the CDSS produces a pop up: *“XYZ syndrome?”* and produces all orders for further diagnostic work up and treatment. You can accept or ignore each order. (4G)
- Using NLP to explore your patient history and clinical examination, in combination with lab and imaging results, the CDSS produces a pop up: *“XYZ syndrome?”* with a short explanation on why this seems a plausible diagnosis (4H)
- Using NLP to explore your patient history and clinical examination, in combination with lab and imaging results, the CDSS produces a pop up: *“XYZ syndrome?”* with a link to the most recent guidelines of the European XYZ association (4I)
